# A Potential Immune-Related Long Non-coding RNA Prognostic Signature for Ovarian Cancer

**DOI:** 10.3389/fgene.2021.694009

**Published:** 2021-07-21

**Authors:** Xue Pan, Fangfang Bi

**Affiliations:** Department of Obstetrics and Gynecology, Shengjing Hospital of China Medical University, Shenyang, China

**Keywords:** ovarian cancer, immune-related lncRNAs, ceRNA network, prognostic signature, The Cancer Genome Atlas, Gene Expression Omnibus

## Abstract

Ovarian cancer (OC), the most lethal gynecologic malignancy, ranks fifth in cancer deaths among women, largely because of late diagnosis. Recent studies suggest that the expression levels of immune-related long non-coding RNAs (lncRNAs) play a significant role in the prognosis of OC; however, the potential of immune-related lncRNAs as prognostic factors in OC remains unexplored. In this study, we aimed to identify a potential immune-related lncRNA prognostic signature for OC patients. We used RNA sequencing and clinical data from The Cancer Genome Atlas and the Gene Expression Omnibus database to identify immune-related lncRNAs that could serve as useful biomarkers for OC diagnosis and prognosis. Univariate Cox regression analysis was used to identify the immune-related lncRNAs with prognostic value. Functional annotation of the data was performed through the GenCLiP310 website. Seven differentially expressed lncRNAs (AC007406.4, AC008750.1, AL022341.2, AL133351.1, FAM74A7, LINC02229, and HOXB-AS2) were found to be independent prognostic factors for OC patients. The Kaplan-Meier curve indicated that patients in the high-risk group had a poorer survival outcome than those in the low-risk group. The receiver operating characteristic curve revealed that the predictive potential of the immune-related lncRNA signature for OC was robust. The prognostic signature of the seven lncRNAs was successfully validated in the GSE9891, GSE26193 datasets and our clinical specimens. Multivariate analyses suggested that the signature of the seven lncRNAs was an independent prognostic factor for OC patients. Finally, we constructed a nomogram model and a competing endogenous RNA network related to the lncRNA prognostic signature. In conclusion, our study reveals novel immune-related lncRNAs that may serve as independent prognostic factors in OC.

## Introduction

Ovarian cancer (OC) is one of the most common malignancies of the female reproductive system and a leading cause of cancer-related deaths (Li et al., 2019). Owing to the lack of reliable biomarkers, 70% of OC cases are diagnosed at an advanced stage, and the 5-year survival rate is less than 50% (Lin et al., 2019). OC is mainly treated by cell abatement combined with adjuvant chemotherapy; however, the recurrence and metastasis rates are high, and the overall prognosis is poor. Therefore, there is an urgent need to further explore the pathogenesis and development of OC and find potential tumor prognostic markers or therapeutic targets for improving the prognosis of patients. Immunotherapy has been recently established as a new treatment method. The mechanism of immune escape in OC is correlated with the prognosis of patients (Odunsi, 2017; Kandalaft et al., 2019).

Long non-coding RNAs (lncRNAs) are a group of RNA molecules with transcripts of greater than 200 nucleotides in length; they have a structure similar to that of mRNA and can affect cell growth, development, proliferation, and differentiation at multiple levels, including epigenetic, transcriptional, and post-transcriptional levels (Cao et al., 2019). The function of lncRNAs is related to their subcellular localization. Some lncRNAs are localized in the cytoplasm; they not only interfere with the post-translational modification of proteins and cause abnormal signal transduction but also affect the post-transcriptional expression of mRNAs in the cytoplasm through various mechanisms (e.g., by acting as ceRNAs) (Chen, 2016). Imbalanced expression of some lncRNAs is frequently involved in the progression of numerous malignancies (O’Brien et al., 2021; Wang Y. et al., 2021; Zhang et al., 2021). Recent studies have reported that lncRNAs are directly or indirectly involved in tumor immune regulation through a variety of mechanisms. For example, the expression of LINC00936 was found to be positively correlated with the numbers of CD3^+^ and CD4^+^ cells and negatively correlated with the number of CD8^+^ cells in the peripheral blood of patients with gastric cancer. Downregulated LINC00936 expression was also shown to promote the immune escape and migration of gastric cancer cells (Li et al., 2021). In addition, the lncRNA PCAT6 could induce M2 polarization of macrophages in the peripheral blood of patients with cholangiocarcinoma, thus promoting malignancy (Tu et al., 2020). Moreover, the downregulation of LNC-290 expression was found to inhibit LPS-induced B cell proliferation, activation, and differentiation by blocking the LPS/TLR4 signaling pathway (Wang F. et al., 2021).

Numerous studies have focused on the establishment of tumor prognosis prediction models using a signature based on the expression levels of immune-related lncRNAs. A recent study (Zhou et al., 2021) identified a lncRNA signature related to tumor-infiltrating B lymphocytes, which could predict prognosis and directed immunotherapy of patients with bladder cancer. Another study (Kong et al., 2020) reported the use of a prognostic model comprising two immune-related lncRNAs to predict the prognosis of hepatocellular carcinoma. Further, a tumor immune infiltration-associated lncRNA signature was developed to improve the prognosis and immunotherapy response of non-small cell lung cancer (Sun et al., 2020). Zhou et al. (2018) reported a prognostic model comprising six immune-related lncRNAs for glioblastoma multiforme. Zhou et al. (2017) also discovered immune-related lncRNAs to predict the prognosis of diffuse large B cell lymphoma. In this study, we screened immune-related lncRNAs to predict the prognosis of OC, based on a series of bioinformatic approaches. Our study findings might be valuable in identifying potential prognostic markers and therapeutic targets for OC.

## Materials and Methods

### Data Acquisition

Ovarian cancer-related RNA sequences (RNA-seq) and clinical data from The Cancer Genome Atlas (TCGA) database^1^ were downloaded and included in a training group. Meanwhile, GSE9891 and GSE26193 datasets downloaded from the Gene Expression Omnibus (GEO) database^2^ were included in validation groups. TCGA-OV dataset included 379 OC patients, whereas the GSE9891 (Tothill et al., 2008) and GSE26193 (Mieulet et al., 2021) datasets included 285 and 107 OC patients, respectively. Perl was used for data integration and extraction of lncRNA expression and corresponding clinical data. Regarding the clinical data, extracted items included patient number, survival time, survival status, age, stage, grade, vascular metastasis, and lymphatic metastasis.

### Specimen Collection

Sixty OC samples were collected from January to December 2015 at ShengJing Hospital of China Medical University (Shenyang, China) and included in the experimental validation dataset. The inclusion criteria were as follows: (1) High-grade serous OC diagnosed by post-operative pathology; (2) Absence of chemotherapy, radiotherapy, immunotherapy, and other treatments before surgery; (3) No history of other tumors or ovary-related diseases. Clinical information of the OC samples is presented in Supplementary Table 1. This study was approved by the ethics committee of the ShengJing Hospital of China Medical University, and informed consent was obtained from all patients.

### Identification of Immune-Related lncRNAs

A total of 2499 immune-related genes (IRGs) were downloaded from the ImmPort database.^3^ The package “corrplot” in R software was used to calculate the correlation between the extracted lncRNAs from TCGA-OV dataset and IRGs to screen out immune-related lncRNAs based on the screening criteria *p* < 0.001 and |*R*| > 0.4, by Pearson correlation analysis (Li et al., 2020).

### Establishment of a Risk-Score Model Based on the Immune-Related lncRNAs

Immune-related lncRNA data and corresponding clinical data of OC patients were used to establish a prognostic model. First, univariate Cox regression analysis was used to evaluate the relationship between the expression levels of immune-related lncRNAs and overall survival (OS). The immune-related lncRNAs associated with the prognosis of OC were screened using *P* < 0.001 as the screening criterion. Least absolute shrinkage and selection operator (LASSO) regression and stepwise regression analyses were used to further narrow down the prognosis-related lncRNAs selected by univariate Cox regression analysis. The screened lncRNAs were analyzed via multivariate Cox regression, and the regression coefficients were obtained. Finally, we established a risk-score model based on the expression of selected immune-related lncRNAs and calculated a risk score for each OC patient using the following formula: risk score = exp1 × β1 + exp2 × β2 … + expn × βn (expn represents the expression value of each lncRNA, and βn represents the regression coefficient) (Li and Meng, 2019).

### Real-Time qPCR

Real-time qPCR was used to detect the relative expression levels of selected immune-related lncRNAs in the 60 OC tissues. Total RNA of OC samples was extracted using TriZol Reagent (Invitrogen, United States). cDNA synthesis was carried out by adding 2 μg total RNA in a 20 μL system according to the AMV reverse transcriptase reagent box. Real-time PCR was performed using a 2 × SYBR Green PCR Master Mix, with appropriate amounts of cDNA as a template, primer concentration of 0.4 μmol/L, and a 15 μL mixture for amplification. Three parallel samples were set for each sample to be tested, and corresponding upstream and downstream primers were designed and synthesized according to the target gene for PCR amplification. Next, the 2^–ΔΔ*Ct*^ method was performed to calculate the relative gene expression with U6 serving as an internal reference. The sequences of primers used for RT-qPCR are presented in Supplementary Table 2.

### Evaluation and Validation of the Risk-Score Model

Each patient was assigned a corresponding risk score using the risk-score model. We then classified patients into high- and low-risk groups, using the median risk score as a cut-off. The Kaplan-Meier (K-M) method was used to compare the survival outcomes between these two groups. A receiver operating characteristic (ROC) curve was plotted to evaluate the predictive value of the risk-score model. To evaluate this model further, we validated the risk-score model in the GSE9891 and GSE26193 datasets and our clinical specimens based on the expression levels of selected immune-related lncRNAs, using the same method as in TCGA dataset. K-M and ROC curves were plotted to evaluate the prognostic prediction efficiency in the validation model. Finally, we performed univariate and multivariate Cox regression analyses, based on the risk score and other clinical features, to determine whether the risk score was an independent prognostic factor in both testing and validation datasets.

### Construction of a Nomogram Model

We constructed a nomogram model based on the expression levels of the selected immune-related lncRNAs using the “rms” package in R software. Calibration curves were drawn to assess the consistency between actual and predicted survival rates. In the nomogram, the appropriate point on the corresponding coordinate axis is identified with reference to the variables of the patient through which a vertical line is drawn. The point where this line intersects with the fractional axis is the score of the variables, and the sum of the scores of each variable is the total score. In the same way, the value of the total score is read on the survival axis, which is the probability of a patient surviving in a given period of time (Hou et al., 2019; Yu and Zhang, 2019).

### Construction of a ceRNA Network

A competing endogenous RNA (ceRNA) network was constructed based on the selected immune-related lncRNAs and the corresponding IRGs. First, the miRDB website^4^ was used to identify the miRNAs that interacted with the selected immune-related lncRNAs (Wong and Wang, 2015). We obtained the sequence of lncRNA from UCSC^5^. Then, select ‘‘Custom Prediction’’ on miRDB, and input the lncRNA sequence to obtain the predicted miRNAs. Next, the interactions between IRGs and possible miRNAs were predicted using the miRWalk website^6^ (Sztromwasser et al., 2021). After identifying the miRNAs that overlapped between the miRDB and miRWalk website, the Cytoscape software was used to visualize the lncRNA-miRNA-mRNA ceRNA network. Finally, the GenCLiP310 website^7^ was used to perform functional enrichment analysis to explore the functions of the genes in this ceRNA network (Liang et al., 2020).

## Results

### Establishment of a Risk-Score Model Based on the Immune-Related lncRNAs for OC Patients

**FIGURE 1:**
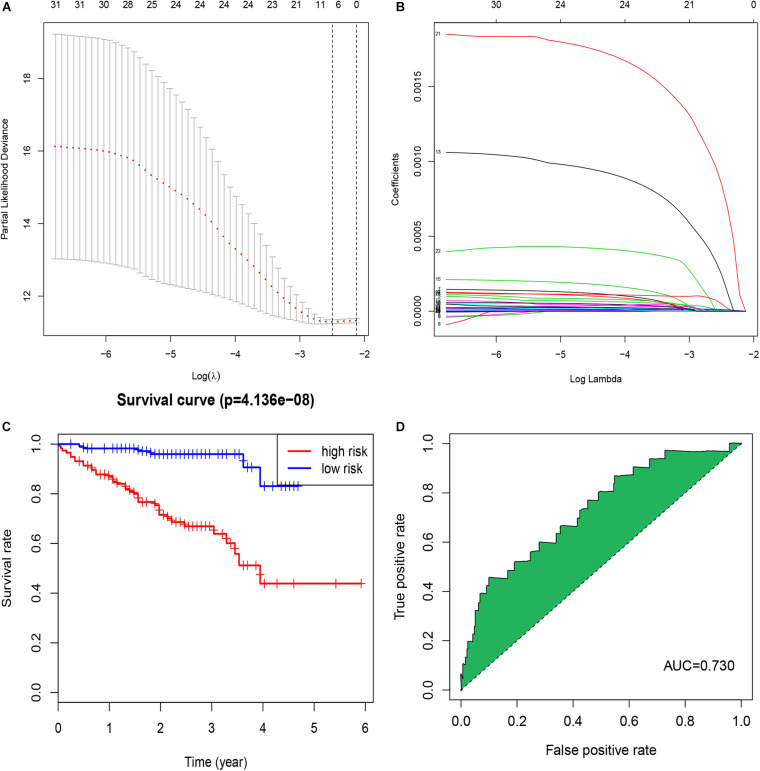
Identification of a signature of seven immune-related lncRNAs. **(A)** The penalization coefficient λ in the LASSO model was tuned using 10-fold cross-validation and the minimum criterion. AUC metrics (*y*-axis) were plotted against log(λ) (bottom *x*-axis). The top x-axis indicates the number of predictors for the given log (λ). Red dots indicate the average AUC for each model at the given λ, and vertical bars through the red dots show the upper and lower values of the AUC, according to the 10-fold cross-validation. The black dotted line on the left defines the optimal λ (where the model provides its best fit to the data). For the optimal λ, seven immune-related lncRNAs with non-zero coefficients were selected. **(B)** LASSO coefficient profiles of the 32 immune-related lncRNAs. **(C)** The K-M survival curve shows the results of survival analysis for the signature of seven immune-related lncRNAs in TCGA-OV dataset. **(D)** ROC curve analysis of the signature of seven immune-related lncRNAs in TCGA-OV dataset.

A total of 42,727 immune-related lncRNAs were screened by correlation analysis in R software based on the RNA sequences in TCGA-OV dataset. Univariate Cox regression was used to screen the immune-related lncRNAs with a significant prognostic value for OC patients; in total, 32 immune-related lncRNAs were selected (*p* < 0.001, Table 1). LASSO Cox regression analysis was used to further screen out the immune-related lncRNAs closely related to the prognosis of OC patients, of which seven lncRNAs (AC007406.4, AC008750.1, AL022341.2, AL133351.1, FAM74A7, LINC02229, and HOXB-AS2) were selected (Figures 1A,B). These lncRNAs were included in multivariate Cox regression analysis, and regression coefficients of each lncRNA were calculated (Table 2). The prognostic model formula obtained to evaluate the risk score of each patient was as follows: risk score = 0.0000229 × expAC007406.4 + 0.0000286 × expAC 008750.1 + 0.0000206 × expAL022341.2 + 0.000779326 × expAL133351.1 + 0.001846185 × expFAM74A7 + 0.000228616 × expLI NC02229 + 0.00000767 × expHOXB-AS2.

**TABLE 1:** The 32 immune-related lncRNAs with a significant prognostic value.

id	HR	HR.95L	HR.95H	*p* value
AC007786.3	1.000059	1.000034	1.000085	5.41E-06
HID1-AS1	1.000014	1.000008	1.00002	5.52E-06
AL359636.2	1.000004	1.000002	1.000006	5.62E-06
FAM74A7	1.002269	1.00123	1.003309	1.84E-05
OVAAL	1.00002	1.000011	1.000029	2.60E-05
AL022341.2	1.000038	1.00002	1.000057	4.51E-05
LINC02578	1.000003	1.000001	1.000004	7.42E-05
AL050404.1	1.000224	1.000111	1.000336	0.000101
AC092994.1	1.000603	1.000298	1.000908	0.000108
LINC01484	1.000007	1.000003	1.00001	0.000115
AL133351.1	1.001118	1.000544	1.001692	0.000135
AL136369.2	1.000162	1.000079	1.000245	0.000136
CELF2-DT	1.00021	1.0001	1.00032	0.000193
AL022313.2	1.000002	1.000001	1.000004	0.000201
AL596218.1	1.00005	1.000023	1.000077	0.000228
LINC02238	1.000106	1.00005	1.000163	0.000237
HAO2-IT1	1.001005	1.000463	1.001547	0.000276
LINC02854	1.000064	1.000029	1.000098	0.000283
HOXB-AS2	1.000009	1.000004	1.000014	0.000292
AL450322.2	1.00005	1.000023	1.000077	0.000342
AC016747.2	1.000008	1.000004	1.000013	0.000404
AL161431.1	1.000001	1	1.000001	0.000408
AC034114.2	1.000132	1.000059	1.000205	0.000416
LINC01109	1.000016	1.000007	1.000025	0.000521
AC007406.4	1.000031	1.000013	1.000049	0.00055
AC007786.2	1.000013	1.000005	1.00002	0.000551
LINC02229	1.000191	1.000082	1.0003	0.000577
AL160237.1	1.000157	1.000065	1.000248	0.000802
AC008750.1	1.000034	1.000014	1.000053	0.000831
LINC02605	1.000061	1.000025	1.000097	0.000881
AC027309.1	1.000079	1.000032	1.000126	0.000929
AC245123.1	1.000431	1.000174	1.000687	0.000999

**TABLE 2:** Regression coefficients of the seven immune-related lncRNAs.

id	coef	HR	HR.95L	HR.95H	*p* value
AC007406.4	2.29E-05	1.000023	1.000004	1.000042	0.020325
AC008750.1	2.86E-05	1.000029	1.000007	1.00005	0.007983
AL022341.2	2.06E-05	1.000021	0.999999	1.000042	0.063851
AL133351.1	0.000779	1.00078	1.000147	1.001413	0.015758
FAM74A7	0.001846	1.001848	1.000681	1.003016	0.001903
LINC02229	0.000229	1.000229	1.000122	1.000335	2.61E-05
HOXB-AS2	7.67E-06	1.000008	1.000002	1.000013	0.005613

### Evaluation and Validation of the Risk-Score Model

**FIGURE 2:**
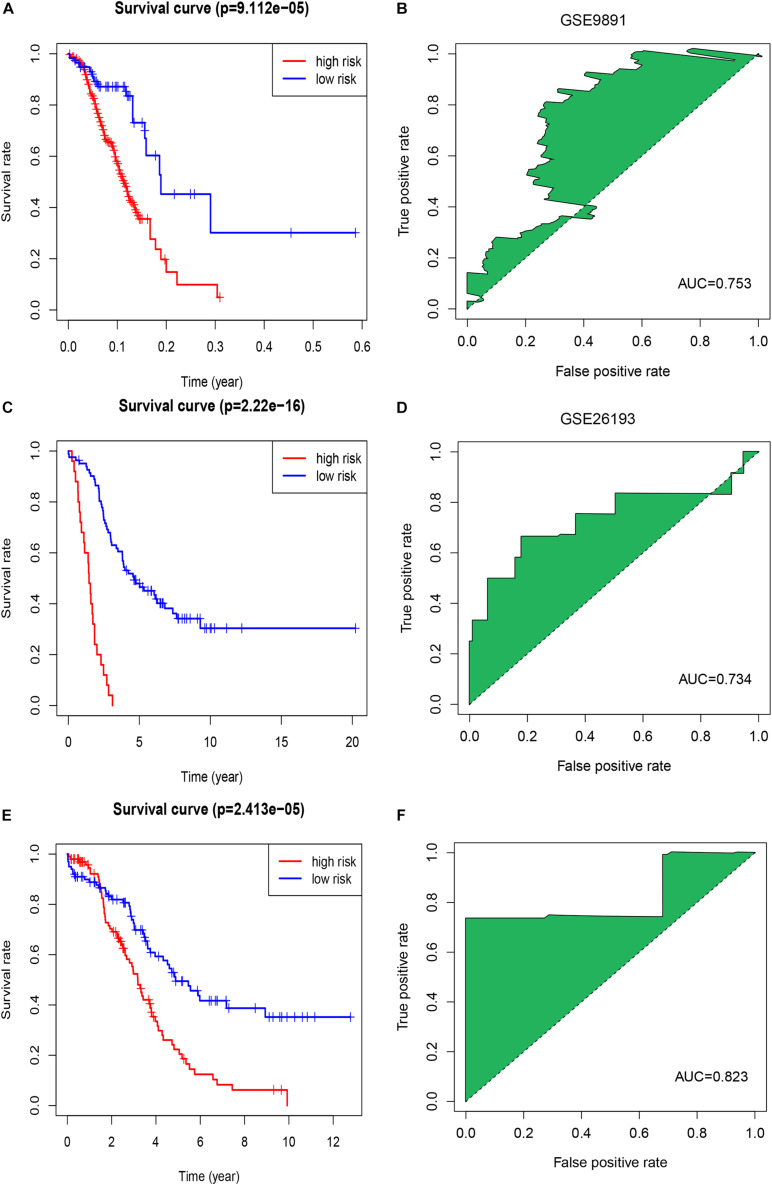
Evaluation of the signature of seven immune-related lncRNAs in the GSE9891 and GSE26193 datasets. **(A)** The K-M survival curve shows the results of survival analysis for the signature of seven immune-related lncRNAs in the GSE9891 dataset. **(B)** ROC curve analysis of the signature of seven immune-related lncRNAs in the GSE9891 dataset. **(C)** The K-M survival curve shows results of survival analysis for the signature of seven immune-related lncRNAs in the GSE26193 dataset. **(D)** ROC curve analysis of the signature of seven immune-related lncRNAs in the GSE26193 dataset. **(E)** The K-M survival curve shows the results of survival analysis for the signature of seven immune-related lncRNAs in our clinical specimens. **(F)** ROC curve analysis of the signature of seven immune-related lncRNAs in our clinical specimens.

We calculated a risk score for each patient using the risk-score model and divided the patients in TCGA-OV dataset into high- and low-risk groups, using the median risk score as a cut-off. The K-M curve indicated that patients in the high-risk group had a poorer outcome than those in the low-risk group (Figure 1C). The accuracy of the risk score in predicting OS was evaluated using a ROC curve; the area under the curve (AUC) value was 0.73 (Figure 1D), suggesting that the prognostic ability of the risk-score model was high. We then validated the risk-score model in the GSE9891 and GSE26193 datasets and our clinical specimens based on the expression level of the seven immune-related lncRNAs. Corresponding K-M curves also indicated that the risk score was strongly correlated with a poor outcome (Figures 2A,C,E). The AUC values of the ROC curves for the GSE9891 and GSE26193 datasets and our clinical specimens were 0.753 (Figure 2B), 0.734 (Figure 2D), and 0.823 (Figure 2F), respectively, highlighting the robust predictive potential of the risk-score model for OC patients.

### The Risk Score May Be an Independent Prognostic Factor for OC Patients

**FIGURE 3:**
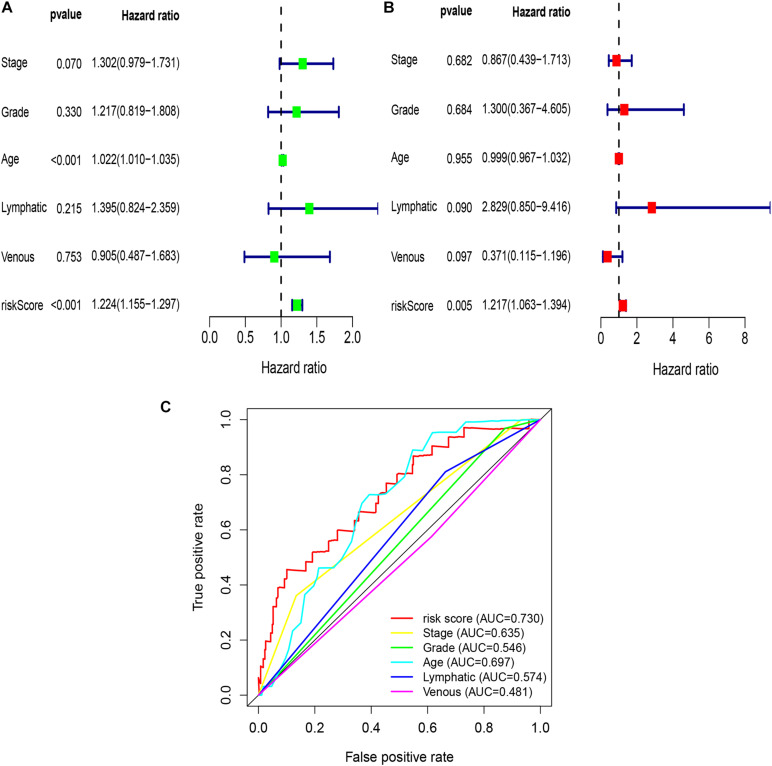
Univariate and multivariate Cox regression analyses based on the risk score and other clinical features in TCGA-OV dataset. **(A)** Univariate Cox regression analyses. **(B)** Multivariate Cox regression analyses. **(C)** ROC curve analysis of the risk score and other clinical features.

**FIGURE 4:**
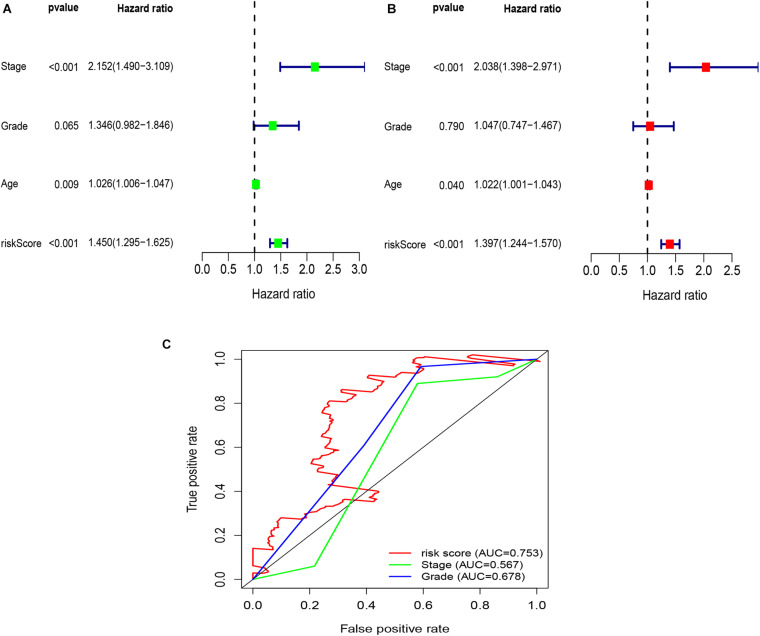
Univariate and multivariate Cox regression analyses based on the risk score and other clinical features in the GSE9891 dataset. **(A)** Univariate Cox regression analyses. **(B)** Multivariate Cox regression analyses. **(C)** ROC curve analysis of the risk score and other clinical features.

**FIGURE 5:**
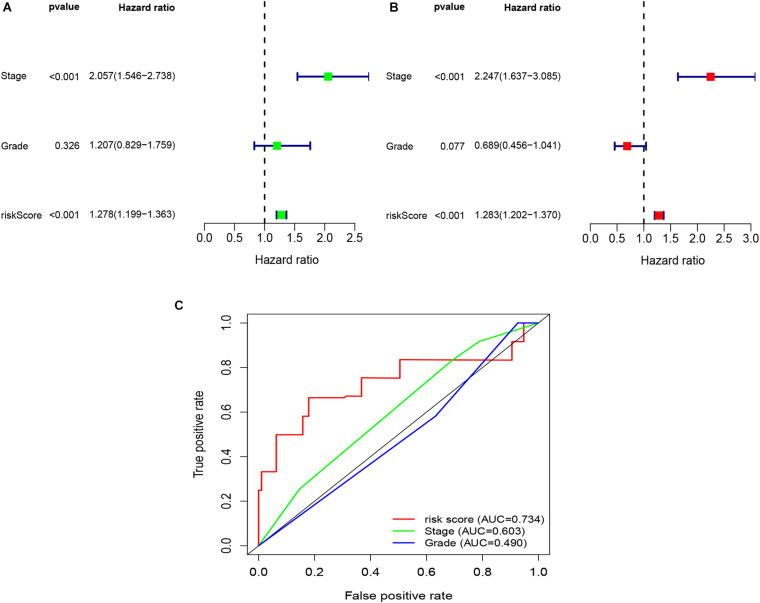
Univariate and multivariate Cox regression analyses based on the risk score and other clinical features in the GSE26193 dataset. **(A)** Univariate Cox regression analyses. **(B)** Multivariate Cox regression analyses. **(C)** ROC curve analysis of the risk score and other clinical features.

**FIGURE 6:**
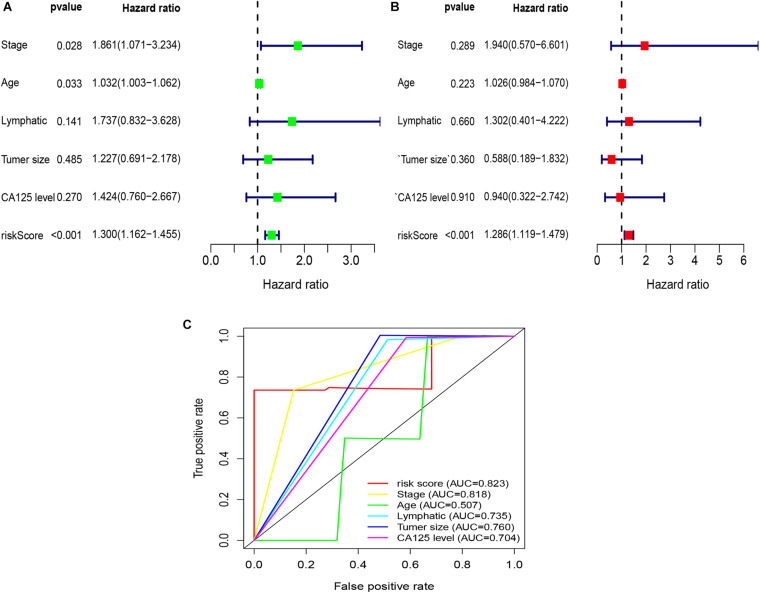
Univariate and multivariate Cox regression analyses based on the risk score and other clinical features in our clinical specimens. **(A)** Univariate Cox regression analyses. **(B)** Multivariate Cox regression analyses. **(C)** ROC curve analysis of the risk score and other clinical features.

To explore whether the risk score was an independent prognostic factor for OC patients, univariate and multivariate Cox regression analyses were performed. In TCGA-OV dataset, univariate Cox regression analyses indicated that age and risk score were related to the prognosis of OC patients (Figure 3A), while multivariate Cox regression analyses revealed that only the risk score was an independent prognostic factor for OC patients (Figure 3B). In the GSE9891 dataset, the *p*-values for age, stage, and risk score were less than 0.05 in both univariate and multivariate Cox regression analyses (Figures 4A,B). In the GSE26193 dataset, univariate and multivariate Cox regression analyses revealed that the stage and risk score had statistical significance in predicting the prognosis of OC patients (Figures 5A,B). We found that the *p*-values of risk score were less than 0.001 in both univariate and multivariate Cox regression analyses (Figures 6A,B). Finally, we plotted the ROC curve to compare the prognostic power of the risk score with other clinical information in the training and validation groups. We found that the prognostic power of the risk score was higher than that of the other clinical parameters (Figures 3C, 4C, 5C, 6C).

### Construction of a Nomogram Model

**FIGURE 7:**
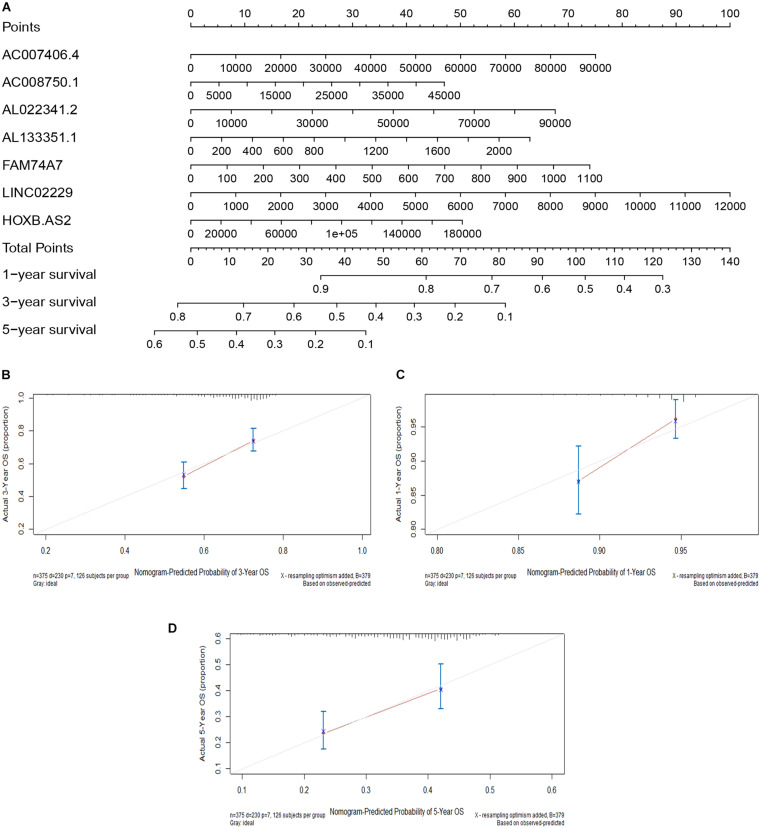
Construction of a nomogram model. **(A)** A nomogram for predicting the 1-, 3-, and 5-year overall survival rates of OC patients. **(B)** The calibration curve at 1 year. **(C)** The calibration curve at 3 years. **(D)** The calibration curve at 5 years.

We constructed a nomogram model based on the expression levels of the seven immune-related lncRNAs to predict the survival rates of OC patients at 1, 3, and 5 years (Figure 7A). The calibration curves at 1, 3, and 5 years revealed high consistency between the actual and predicted survival rates, suggesting the powerful predictive performance of the nomogram model (Figures 7B–D).

### Construction of a ceRNA Network

**FIGURE 8:**
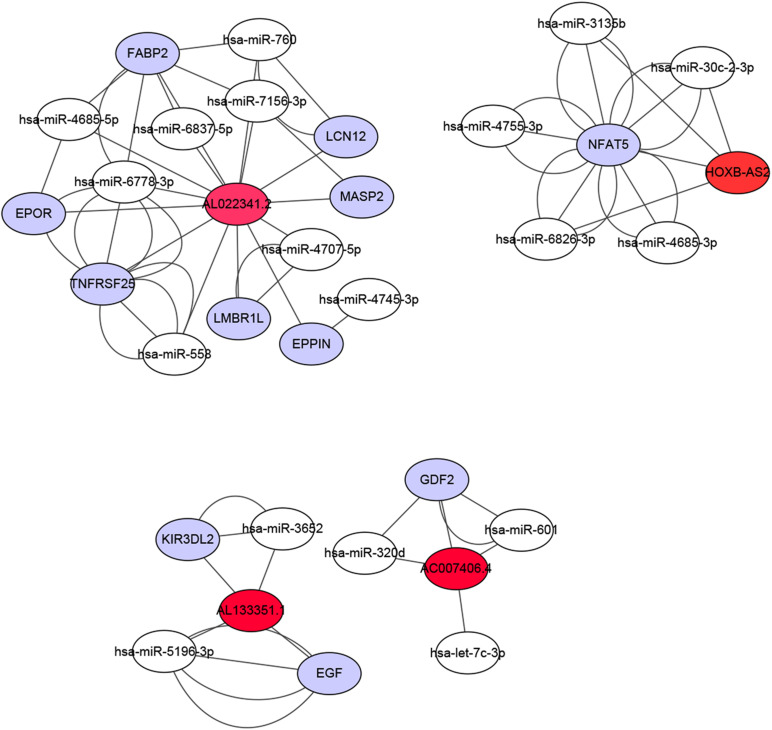
Construction of a ceRNA network associated with the immune-related lncRNAs. The ceRNA network included four immune-related lncRNAs, 11 immune-related genes, and 18 miRNAs. Red represents lncRNA, white represents miRNA, and purple represents mRNA.

**FIGURE 9:**
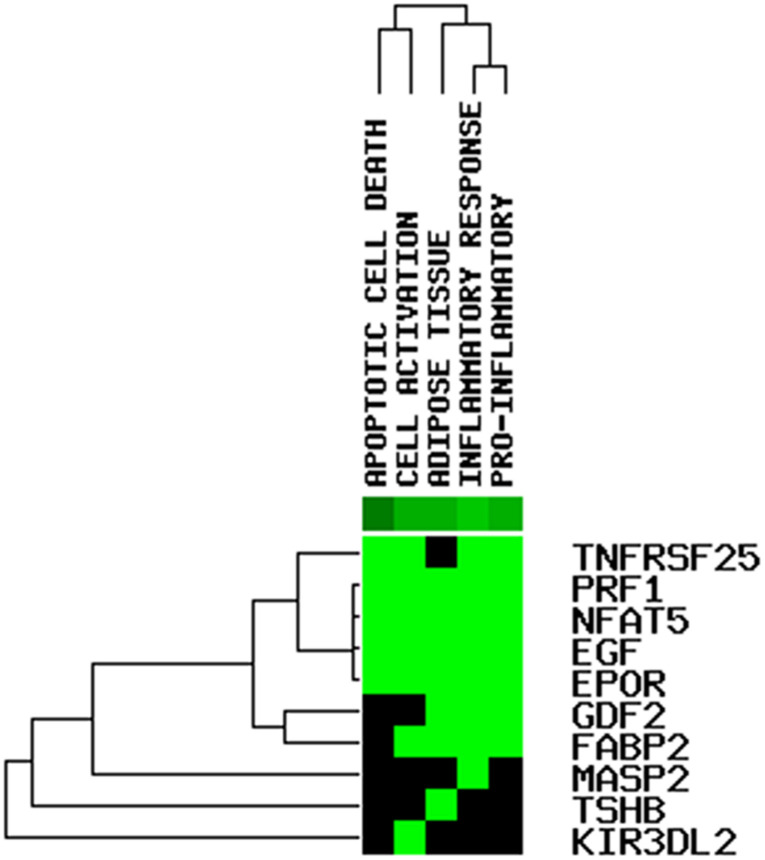
Functional enrichment analysis on the mRNAs in the ceRNA network.

We constructed a ceRNA network based on the seven immune-related lncRNAs and 18 corresponding IRGs (Table 3). microRNAs (miRNAs) that interacted with the seven selected immune-related lncRNAs were obtained from the miRDB website, followed by miRNAs interacting with the 18 IRGs from the miRWalk website. After identifying the miRNAs that overlapped between these two groups, a ceRNA network, including four immune-related lncRNAs, 11 IRGs, and 18 miRNAs, was obtained and visualized using Cytoscape software (Figure 8). Finally, the 18 IRGs were input into the GenCLiP310 website to explore the functions of these genes via functional enrichment analysis; the genes were mainly involved in apoptotic cell death, cell activation, adipose tissue, inflammatory response, and pro-inflammatory functions (Figure 9).

**TABLE 3:** The seven immune-related lncRNAs and 18 corresponding immune-related genes.

GENE1	GENE2	P	R
AC007406.4	GDF2	3.01E-31	0.549234
AC008750.1	PRF1	3.54E-16	0.402308
AL022341.2	DEFB107B	4.97E-16	0.400455
AL022341.2	LCN12	4.21E-33	0.563207
AL022341.2	LMBR1L	1.13E-21	0.464443
AL022341.2	FABP2	2.79E-16	0.403608
AL022341.2	EPPIN	4.91E-21	0.457836
AL022341.2	MASP2	2.22E-21	0.461423
AL022341.2	EPOR	8.28E-25	0.494884
AL022341.2	TNFRSF25	8.18E-29	0.529823
AL133351.1	KIR3DL2	5.29E-19	0.435841
AL133351.1	EGF	3.84E-29	0.532518
FAM74A7	TRAV3	5.64E-19	0.435521
FAM74A7	TRAV8-2	9.87E-21	0.454657
FAM74A7	TRAV8-3	5.61E-25	0.496444
HOXB-AS2	NFAT5	3.07E-19	0.438486
HOXB-AS2	TSHB	1.18E-20	0.453826
LINC02229	TRAJ31	3.02E-20	0.449487

## Discussion

Investigation of the molecular mechanisms underlying OC pathogenesis is important for the early diagnosis, treatment, and improved prognosis of OC. LncRNAs play a role in the promotion or inhibition of tumor growth through a variety of molecular mechanisms. Many lncRNAs are involved in tumor immune responses. Therefore, understanding the basic mechanism underlying immune-related lncRNA regulation may provide useful insights for the development of novel cancer treatments.

In this study, online datasets were used to determine a new and effective immune-related lncRNA prognosis signature for OC. This signature may affect the immune-related lncRNA status of OC patients and provide potential biomarkers for clinical therapeutic intervention.

In this study, we performed a comprehensive analysis of immune-related lncRNAs and obtained OC RNA-seq and clinical data from TCGA and GEO databases. First, we obtained a signature of seven immune-related lncRNAs with a prognostic value via univariate and multivariate Cox regression analysis. The seven differentially expressed lncRNAs (AC007406.4, AC008750.1, AL022341.2, AL133351.1, FAM74A7, LINC02229, and HOXB-AS2) were found to be independent prognostic factors for OC. The K-M curve revealed that patients in the low-risk group had a longer OS than those in the high-risk group. The ROC curve suggested that the predictive potential of the risk-score model for OC patients was robust. In benchmark comparisons, the AUC (0.703) was comparable to or better than that of other published gene signatures (AUC: 0.604–0.813) (Zhou et al., 2016; Yang et al., 2017; Meng et al., 2020; Zhao et al., 2021). Univariate and multivariate Cox regression analyses indicated that the risk score was an independent prognostic factor for OC. Finally, the results of our analysis were successfully verified in the GSE9891 and GSE26193 datasets and our clinical specimens.

The signature of seven immune-related lncRNAs (AC007406.4, AC008750.1, AL022341.2, AL133351.1, FAM74A7, LINC02229, and HOXB-AS2) was associated with the poor prognosis of OC patients. Among these immune-related lncRNAs, the functions of AC007406.4, AL022341.2, AL133351.1, FAM74A7, and LINC02229 were not reported previously. However, Sage et al. (2020) investigated the relationship between AC008750.1 and NK cells. In activated NK cells, the expression of AC008750.1 was induced, which in turn enhanced the anti-tumor ability of these cells. In addition, (Huang et al., 2020) established an immune score-based risk signature including nine genes for predicting the prognosis of oral squamous cell carcinoma. AC008750.1, one of the nine IRGs, was found to be associated with the poor prognosis of OSCC patients, which is also consistent with our findings. HOXB-AS2 has been reported to be abnormally expressed in the epicardial adipose tissue in atrial fibrillation (Shi et al., 2020). However, its role in tumor tissues has not been revealed. Future studies are needed to validate our results *in vivo* and *in vitro* and to explore the mechanisms of action of these immune-related lncRNAs in OC.

Most known mechanisms of action of lncRNAs involve RNA-RNA and/or RNA-protein interactions. Among them, the mechanism of ceRNAs is particularly important in exploring how lncRNAs participate in the regulation of malignant tumors (Chen et al., 2020; Xiu et al., 2020). lncRNAs can competitively adsorb miRNAs by binding to miRNA response elements, resulting in target gene silencing by inhibiting the binding of miRNA and mRNA. The mechanism of this RNA-RNA interaction is called ceRNA regulation (Ye et al., 2019). We used a series of bioinformatics analyses to establish ceRNA networks associated with the seven immune-related lncRNAs to predict the possible mechanisms underlying the involvement of these lncRNAs in the malignancy of OC. Finally, a ceRNA network including four immune-related lncRNAs, 11 IRGs, and 18 miRNAs was obtained and visualized using Cytoscape software; the genes were mainly involved in apoptotic cell death, cell activation, adipose tissue, inflammatory response, and pro-inflammatory functions.

A limitation of this research should be discussed. The specific mechanisms of action of the seven immune-related lncRNAs involved in the immune regulation of OC have not been explored.

## Conclusion

Using TCGA and GEO, and other bioinformatics methods, we identified a signature of seven immune-related lncRNAs as an independent prognostic factor to predict the prognosis of OC. These seven immune-related lncRNAs may serve as prognostic markers and new therapeutic targets for OC.

## Data Availability Statement

The datasets presented in this study can be found in online repositories. The names of the repository/repositories and accession number(s) can be found in the article/Supplementary Material.

## Ethics Statement

The studies involving human participants were reviewed and approved by this study was approved by the Ethics Committee of the ShengJing Hospital of China Medical University, and informed consent was obtained from all patients. The patients/participants provided their written informed consent to participate in this study.

## Author Contributions

Both authors conceived and designed the study, developed the methodology, analyzed and interpreted the data, wrote, reviewed, revised the manuscript, and approved the submitted version.

## Conflict of Interest

The authors declare that the research was conducted in the absence of any commercial or financial relationships that could be construed as a potential conflict of interest.

## Footnotes

1 https://portal.gdc.cancer.gov/

2 https://www.ncbi.nlm.nih.gov/

3 https://www.immport.org/shared/

4 http://mirdb.org/custom.html

5 http://genome.ucsc.edu/

6 http://mirwalk.umm.uni-heidelberg.de/

7 http://ci.smu.edu.cn/genclip3/analysis.php

## Supplementary Material

Supplementary Table 1 Clinical information of the 60 OC samples collected from ShengJing Hospital of China Medical University.

Supplementary Table 2 RT-qPCR primer sequences.
