# Supplementary material for: A Potential Immune-Related Long Non-coding RNA Prognostic Signature for Ovarian Cancer
Source: Front Genet. 2021 Jul 21;12:694009. doi: 10.3389/fgene.2021.694009 (PMC8335165; doi:10.3389/fgene.2021.694009)
Supplement: Supplementary Table 1 — Clinical information of the 60 OC samples collected from ShengJing Hospital of China Medical University. [file Table_1.DOCX]

| Characteristics | n |
| --- | --- |
| Age(year) |  |
| ≤55 | 28 |
| >55 | 32 |
| Lymph node metastasis |  |
| Positive | 34 |
| Negative | 26 |
| FIGO stage |  |
| I-II | 25 |
| III-IV | 35 |
| Tumer size（cm） |  |
| ≤10 | 36 |
| >10 | 24 |
| CA125 level（U/mL） |  |
| ≤300 | 37 |
| >300 | 23 |
| Survival state |  |
| Alive | 21 |
| Dead | 39 |
