# Supplementary material for: A Potential Immune-Related Long Non-coding RNA Prognostic Signature for Ovarian Cancer
Source: Front Genet. 2021 Jul 21;12:694009. doi: 10.3389/fgene.2021.694009 (PMC8335165; doi:10.3389/fgene.2021.694009)
Supplement: Supplementary Table 2 — RT-qPCR primer sequences. [file Table_2.DOCX]

| Gene | Sequences of the primers |
| --- | --- |
| HOXB-AS2 | F:5' TGGGCTCCTTGGTGGTGATAGATC3'  R:5’ TTCCTGCTTCTCCCTCCCTTTCC3’ |
| LINC02229 | F:5' TGGAGTTTGGTGCCTTCAGTTAGC3’  R:5’ CACGGAAGTATGGAAACACTCACATTC 3’ |
| FAM74A7 | F:5' AGGAGACGTGGAGACAGTTCAGAG 3’  R:5’ ACATCTTCTTGTGGACAGCCTTTGAG 3’ |
| AL133351.1 | F:5' GAACTTTGGACATGGATGGACCTCTC 3’  R:5’ AGTGCTCTTCCTGCCTCAGTCTC 3’ |
| AL022341.2 | F:5’ GCAAGGAATGAGGACTGAGCACTG3’  R:5' GAGGTGGTCGTTCTGGCAAAGG3’ |
| AC008750.1 | F:5’ CACGCCTCCTCTTAACCTACATGATC 3’  R:5' TGGGGCAAAGAGTAGGGTCCTG 3’ |
| AC007406.4 | F:5'GTATTGGTGGGTGGGTGGTTGAC3'  R:5’ TGCTGTGTATGGAGGGATAAAGGAAAC 3’ |
| U6 | F:5’GCTTCGGCAGCACATATACTAAAAT3’  R:5’CGCTTCACGAATTTGCGTGTCAT3’ |
